# Gray plumage color is more cryptic than brown in snowy landscapes in a resident color polymorphic bird

**DOI:** 10.1002/ece3.5914

**Published:** 2020-02-05

**Authors:** Katja Koskenpato, Aleksi Lehikoinen, Carita Lindstedt, Patrik Karell

**Affiliations:** ^1^ The Helsinki Lab of Ornithology Finnish Museum of Natural History University of Helsinki Helsinki Finland; ^2^ Bioeconomy Research Team Novia University of Applied Sciences Ekenäs Finland; ^3^ Department of Biological and Environmental Sciences Centre of Excellence in Biological Interactions University of Jyväskylä Jyväskylä Finland; ^4^ Department of Biology Lund University Lund Sweden

**Keywords:** camouflage, climate change, color polymorphism, *Strix aluco*, survival selection, visual predation

## Abstract

Camouflage may promote fitness of given phenotypes in different environments. The tawny owl (*Strix aluco*) is a color polymorphic species with a gray and brown morph resident in the Western Palearctic. A strong selection pressure against the brown morph during snowy and cold winters has been documented earlier, but the selection mechanisms remain unresolved. Here, we hypothesize that selection favors the gray morph because it is better camouflaged against predators and mobbers in snowy conditions compared to the brown one. We conducted an online citizen science experiment where volunteers were asked to locate a gray or a brown tawny owl specimen from pictures taken in snowy and snowless landscapes. Our results show that the gray morph in snowy landscapes is the hardest to detect whereas the brown morph in snowy landscapes is the easiest to detect. With an avian vision model, we show that, similar to human perceivers, the brown morph is more conspicuous than the gray against coniferous tree trunks for a mobbing passerine. We suggest that with better camouflage, the gray morph may avoid mobbers and predators more efficiently than the brown morph and thus survive better in snowy environments. As winters are getting milder and shorter in the species range, the selection periods against brown coloration may eventually disappear or shift poleward.

## Graphical abstract

Pale gray tawny owl color morph is better camouflaged under snowy landscapes compared to melanistic reddish‐brown morph. This may explain why brown morphs suffer from higher mortality during harsh winter conditions in the north. As winters are getting milder and shorter, the selection periods against brown coloration may eventually disappear or shift poleward.

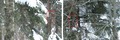

## 1 INTRODUCTION

Predation is one of the most common selection pressures shaping the evolution of animal coloration (Gray & McKinnon, 2007; Nokelainen, Valkonen, Lindstedt, & Mappes, 2014; Punzalan, Rodd, & Hughes, 2005; Willmott, Robinson Willmott, Elias, & Jiggins, 2017). Maybe the most fundamental strategy to gain protection from predators is via camouflage, which makes prey difficult to detect from its background (Merilaita & Stevens, 2011). Efficacy of camouflage is always dependent on its visual background (Endler, 1978; Hughes, Liggins, & Stevens, 2019; Michalis, Scott‐Samuel, Gibson, & Cuthill, 2017). Spatial and temporal variation in visual environments (e.g., darkness of the background) can lead to the evolution of color polymorphisms, that is, the coexistence of genetically different color morphs within a population (Bond & Kamil, 2006; Cook, 2000; Fisher, 1958; Ford, 1945; Roulin, 2004). Color polymorphism can persist in a population if, for example, color morphs have different selective advantages in different environments (Cook, 2000) or predators develop search image for the most common morph, leading to apostatic selection (Bond & Kamil, 2006).

Following the conceptual framework outlined by Merilaita and Stevens (2011), light coloration may provide better camouflage in cold and dry environments in northern higher latitudes whereas darker coloration is likely to be more cryptic in warm and wet environments in lower latitudes. At present, climate change‐driven habitat change may alter these predator–prey interactions, not only by changing species distributions and community structures, but also by affecting the physical and visual properties of an environment. For example, climate change is expected to reduce snow cover period and depth (IPCC, 2014). Thereby, selection for cryptic coloration may be challenged as a consequence of the drastic changes in the landscape and thus visual environment (Atmeh, Andruszkiewicz, & Zub, 2018; Mills et al., 2018; Zimova, Mills, Lukacs, & Mitchell, 2014; Zimova, Mills, & Nowak, 2016).

Selection on coloration due to changes in winter snow conditions has been found in color polymorphic tawny owls (*Strix aluco*). The tawny owl is a resident forest‐dwelling owl species widely spread in the Western Palearctic. Individuals in this species display two heritable color morphs (Brommer, Ahola, & Karstinen, 2005; Karell, Ahola, Karstinen, Valkama, & Brommer, 2011): the pheomelanic reddish‐brown morph (hereafter referred to as “brown morph”) and the less melanic gray morph (Emaresi et al., 2013; Gasparini et al., 2009). In Finland, where tawny owls live in their northernmost range margin, Karell et al. (2011) discovered strong survival selection against the brown morph during cold winters with lots of snow, but this was absent during mild winters with little or no snow. The exact mechanism by which selection against the brown morph in snowy winters was mediated remained unresolved.

Here, we ask if the previously documented variation in selection on coloration under different snow conditions (Karell et al., 2011) is due to differences in camouflage of the color morphs. It has been suggested that variation in light conditions in the habitat is an important factor contributing to the evolution of color polymorphism in birds of prey and owls (Passarotto, Parejo, Penteriani, & Avilés, 2018; San‐José et al., 2019; Tate, Bishop, & Amar, 2016). We hypothesize that the brown morph is more conspicuous than the gray morph in snowy conditions, making it easier to be detected by mobbing passerines and corvids, whereas the difference in crypsis between morphs is less evident in snowless conditions. In the boreal zone, tawny owls spend the daytime roosting in coniferous trees. If they are spotted by mobbing birds—which can also lead to spotting by diurnal predators such as goshawks (*Accipiter gentilis*)—they are forced to move. Mobility becomes energetically costly to the owls, especially during harsh winters when there is little food available to satisfy the increased energy demand.

## 2 MATERIALS AND METHODS

The study was conducted during winter 2017–2018 in Viikki Arboretum, Helsinki, Finland (60°13′N, 25°00′E), which is a typical Finnish tawny owl habitat consisting of cultural landscape and mixed forest. We had access to a gray and a brown mounted tawny owl from the Finnish Museum of Natural History, which we color‐scored using the same scoring method as detailed in Brommer et al. (2005) and Karell, Brommer, Ahola, and Karstinen (2013). This scoring method is based on the amount of pheomelanin pigmentation (evaluated by the researcher) in four different parts of the plumage, resulting in a total color score within the range of 4–14. Scores <10 are categorized gray, and scores of 10 or more are considered brown. The color score of the mounted gray tawny owl used in this study was 4 and that of the brown was 13, making them typical gray and brown tawny owl individuals. We placed the mounted gray and brown owl one at a time on the same branch, and we took identical pictures of them from a given distance and angle in 16 different study spots. The mounted owls were placed perching on Norway spruce (*Picea abies*) or Scots pine (*Pinus sylvestris*), the most common tree species in Finland (Finnish Forest Research Institute, 2014). We took snowless photographs in December and snowy photographs in January, altogether 64 pictures consisting of both color morphs and snowy and snowless landscapes. Pictures were taken with a Canon EOS 550D using a landscape mode in snowless landscapes and normal mode with +1 step exposure value compensation in snowy landscapes. Some pictures were sharpened, and brightness was adjusted with Corel Paint Shop Pro X9 (version 19.2.0.7) to ensure that the repeated pictures were as identical as possible (and only the snow conditions varied). Since the pictures were taken from different angles and distances in 16 different localities, we assumed that the mounted specimens would not be perceived as identical specimens, but rather as typical specimens of the color morph in the different pictures (study pictures available in Dryad).

### 2.1 Human observers

We used a citizen science approach to study the detectability of brown and gray tawny owl morphs in snowy and snowless landscapes. We made eight different picture series out of the whole photoset of 64 pictures, each series consisting of eight photographs. Every series had randomly chosen pictures of both morphs and both landscapes. Pictures taken in the same study plot with different snow conditions were not in the same series.

The use of human observers via online “games” has been recently used in camouflage studies in birds and their eggs (Troscianko, Wilson‐Aggarwal, Griffiths, Spottiswoode, & Stevens, 2017), as well as crabs (Nokelainen, Maynes, Mynott, Price, & Stevens, 2019). Similar citizen science approach has also previously been used to study disruptive coloration with, for example, computer‐generated moth images (Fraser, Callahan, Klassen, & Sherratt, 2007), and detection and learning of camouflage strategies (Troscianko, Lown, Hughes, & Stevens, 2013) and to quantify the appearance of camouflaged prey (Troscianko, Skelhorn, & Stevens, 2017). We designed an online study website where a randomly chosen series of pictures was presented for each volunteer participant. Before the actual survey started, participants were asked to give their e‐mail address, age, and if they had birdwatching as a hobby. We wanted to include a question about birdwatching as hobby (yes/no) to be able to correct for a possible superior search image of birdwatchers as compared to people without potential previous experience of tawny owls. The online study website “https://www.luomus.fi/en/tawny-owl-colour-morphs” was open for survey attempts 29.3.2018–31.5.2018. Before entering the survey, there were introduction and guidelines for participants. The participants were asked to do the survey alone using a computer screen, not a phone or tablet. It was stated that there would be eight pictures altogether in the survey, and if the participant would not find the owl in a given picture within a minute, the program would show the location of the owl after which it was possible to continue to the next picture. The participants were asked to do the survey only once. There was also shown an example of a potential study picture and pictures of the gray and brown mounts used in the study. No personal information (e.g., name) was asked, and the e‐mail address was only used to exclude multiple survey attempts, after which the addresses were discarded. The participants had the mission to find an owl in the pictures as fast as possible. The online system then registered the detection time for every picture. If the owl was not found within 60 s, the system moved to the next picture. Participants did not need to define the color morph of the owls. The online system site was spread via social media and newspapers aiming to maximize participants with different backgrounds to the study.

In total, we got 5,432 survey attempts for the online survey of tawny owl color morphs via the webpage of the Finnish Museum of Natural History. We used the participants' e‐mail addresses as identification to exclude survey attempts conducted multiple times by the same person. We also excluded attempts done with a smartphone or a tablet and also attempts with comments on network or program errors or attempts where the survey was not finished. We used the remaining 5,362 attempts (including 42,896 cases) for analysis.

### 2.2 Validation of the study material

In the online study, we had access to a gray and a brown tawny owl specimen. To validate the use of only one brown and only one gray tawny owl specimen in the study, we conducted an additional survey where volunteers were asked to define the color morph of 10 mounted tawny owls of which two were the mounts used in the online game. With this survey, we aimed to test whether people were able to tell the two morphs apart from a set of specimens with varied plumage coloration, and thus test how well laymen can assign our online game model owls and a set of other tawny owl mounts to their corresponding color morphs. Our hypothesis was that it is as easy to assign color morph to any of the mounted owl as to the model owls. If the two owl mounts used in the online game cannot be distinguished from a pool of gray and brown owl mounts, it indicates that the online game model owls are representative samples of their morph. Six of the mounted owls were provided by the Finnish Museum of Natural History and two by a private taxidermist. For practical reasons (e.g., we could not guarantee that the specimens would remain unharmed), we were not able to take these specimens as part of the main survey conducted in Viikki Arboretum. In addition to these eight mounts, we had the two mounts used in the main survey as part of this validation survey. We took three pictures (one from the back, one from the chest, and one from the side) of the 10 mounted tawny owls against a tree trunk (pictures available in Dryad). We compiled three picture series so that every series had one picture of each mount. The order of appearance of the mounts as well as the angle was randomized in every series. We color‐scored all the mounts according to the method described above where scores range from 4 to 14 (Brommer et al., 2005; Karell et al., 2013). The color scores of the mounts were as follows: 4, 4, 4, 7, 9, 10, 11, 12, 13, and 14, indicating a variation across the range of color score values found in live tawny owls. Color scores 4–9 are defined as gray and scores 10–14 as brown (see Brommer et al., 2005; Karell et al., 2011 for details). We presented one of the three picture series to the volunteers so that each of the 10 pictures was shown one at a time. The task was to compare each of the 10 pictures to pictures of the two mounted tawny owls used in the main survey and to define whether the mounts resemble more the brown or the gray mount in the survey. We then compared the answers to the color definition done by us using the color scoring system. A total of 39 volunteers participated in the survey, resulting in 390 observations as every volunteer defined the morph of 10 specimens.

### 2.3 Validation of the study method

As birds' vision differs from that of humans, for example, birds are able to sense the ultraviolet spectrum of light and humans are not (Cuthill, 2006), we wanted to confirm whether the visibility to human observers is similar to a potential mobbing bird species: a blue tit (*Cyanistes caeruleus*; Bergeron & Fuller, 2018). To validate the method of using human perceivers in this study, we used an avian vision modeling approach. We used the same study specimens as in the human observer experiment and analyzed their conspicuousness to blue tits against different typical natural backgrounds with an avian vision model. To compare the contrasts of the tawny owl plumages against barks and needles of spruce and pine as well as snow, samples of these were photographed with a calibrated Fujifilm FinePix S3 Pro UVIR digital camera recording both ultraviolet (UV image) and human visible wavelengths (human visible image). The camera was equipped with an UV‐transmitting lens (Coastal Optical Systems). For the UV images, we used an UV pass filter (Baader U; 320–380 nm transmittance) and for the human visible images a filter blocking UV and infrared (Baader UV/IR Cut; 400–680 nm transmittance). The photographing was conducted outside under natural light conditions on a snowy and cloudy day in March 2018. The variability of natural light conditions was controlled with a 50% white‐gray standard.

We used the Multispectral Image Calibration and Analysis Toolbox (micaToolbox) (Troscianko & Stevens, 2015) following the established methodology and procedure as explained in, for example, Koski et al. (2017). With micaToolbox, we first combined human visible images and UV images to normalized and linearized multispectral images. We then chose and analyzed regions of interest (three similar‐sized samples per focal pattern located approximately in the same spots in each owl specimen) and converted them to cone‐catch data (Hart, 2001; Hart, Partridge, & Cuthill, 2000; Troscianko & Stevens, 2015). These cone‐catch data were then used for the discrimination model to estimate whether blue tits can discriminate between tawny owl plumage and the coniferous barks and needles. In the color discrimination model, a Weber fraction of 0.05 was used for the most abundant cone type and the relative proportions of cone types in the blue tit retina (longwave = 0.96, mediumwave = 1, shortwave = 0.85, and ultraviolet sensitive = 0.46). A Weber fraction 0.05 was also used for modeling luminance discrimination using the double cones (Sandre, Stevens, & Mappes, 2010; Siddiqi, Cronin, Loew, Vorobyev, & Summers, 2004). The avian vision model uses units called just noticeable differences (JNDs) where values <1–3 indicate that the two colors are likely indistinguishable under optimal light conditions and values >3 indicate that two objects are likely discriminable (Siddiqi et al., 2004).

### 2.4 Statistics

All the statistics were conducted in R version 3.4.4 (R Core Team, 2017). We used a generalized linear mixed model (GLMM) with binomial error distribution and a linear mixed‐effect model (LME) to explain detection probability (found or not) and detection time (in seconds), respectively. We included six fixed effects in the models: snow (categorical: yes/no), color morph of the owl (categorical: brown/gray), age of the volunteers (linear and quadratic effect), birdwatching hobby (categorical: yes/no), rank of the picture within a picture series (linear: 1–8), and snow by color morph interaction. Participant ID and picture series were set as random effects. We used only observations of participants of the age 8–80 since it was obvious that the youngest and the oldest ages given were not the real ages. Continuous variables were standardized to mean value ±1 *SD*. In case of detection time analyses, we log‐transformed the time in order to normalize the data. We present the global models with all effects, and the significance of the fixed variables is tested with a *t* test. GLMM and LME analyses were conducted using functions glmer and lmer (packages of lme4 and lmerTest; Bates, Maechler, Bolker, & Walker, 2015; Kuznetsova, Brockhoff, & Christensen, 2017).

We used a GLMM with binomial error distribution to test whether the observers were more likely to correctly define the color of the two mounts used in the main survey compared to the others (categorical: included in main survey/not included). We also included four additional fixed effects in the models: color score of the owl defined by us using the color scoring method (numeric), angle (categorical: chest/back/side), picture series (categorical: 1/2/3), and rank of the picture within a picture series (linear: 1–10). Participant ID and owl mount ID were set as random effects. The model was conducted using function glmmTMB (package of glmmTMB; Brooks et al., 2017).

## 3 RESULTS

### 3.1 Detection probability of the tawny owl morphs

**Figure 1:**
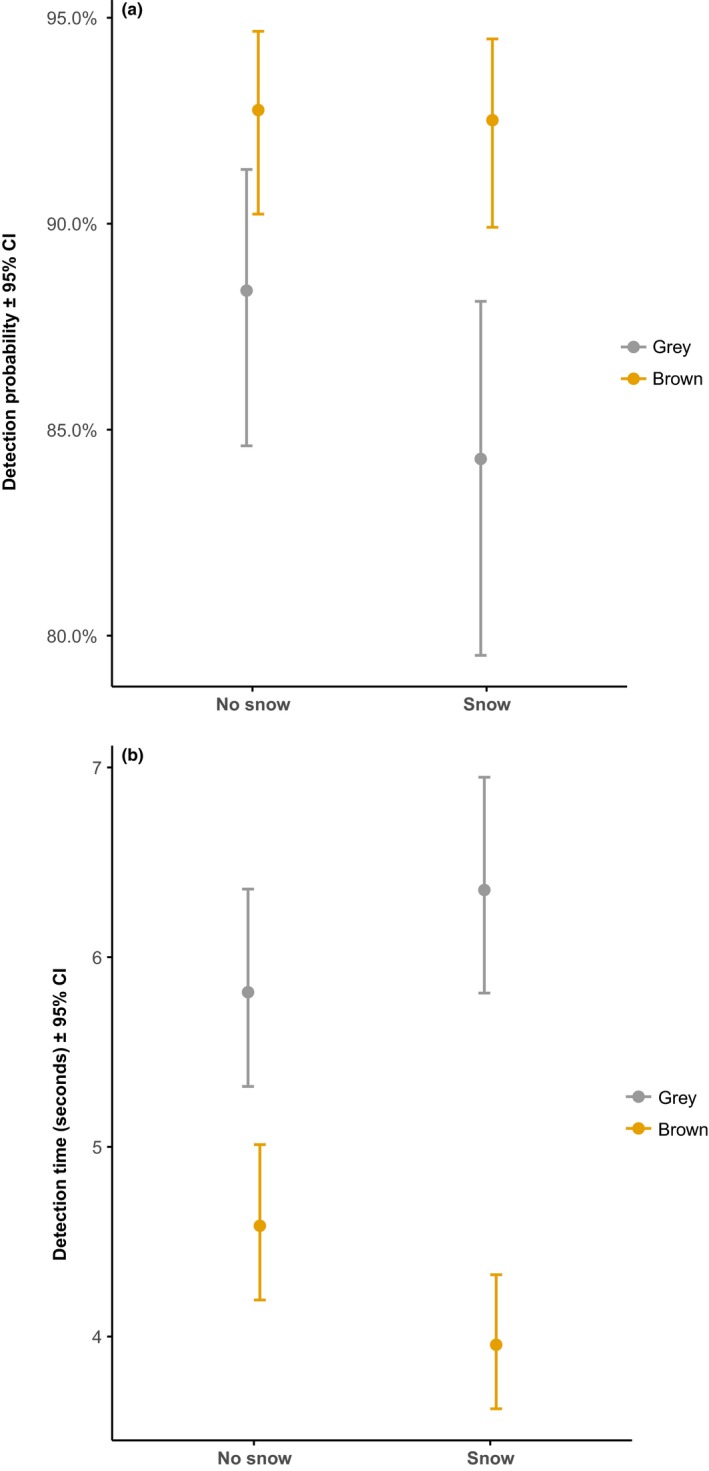
Least squares means with 95% confidence intervals of (a) detection probability and (b) detection time for both color morphs in snowy and snowless landscapes

In 37,564 cases (88%), the owl was found, and in 5,332 cases (12%), the owl was not found in the picture within the 60 s. All fixed effects were significant in the GLMM (Table 1). The owls were easier to find (more often detected) in snowless landscapes than in snowy landscapes. The brown morph was easier to find than the gray morph. The interaction snow by color had a strong effect, where the brown morph in snowy landscapes was easier to detect than the gray morph in snowy landscapes (Figure 1a). Younger participants found owls easier than older participants. Owls became harder to find toward the end of the series of the eight pictures. Birdwatchers found owls more often than non‐birdwatchers.

**Table 1:** GLMM statistics of a model explaining detectability of owls

	Estimate	*SE*	*z*	*p*
Intercept	2.030	0.165	12.28	<.001
Snow (compared to no snow)	–0.351	0.040	–9.02	<.001
Color (brown compared to gray)	0.519	0.045	11.45	<.001
Color by snow (brown in snow compared to gray in snow)	0.316	0.063	5.03	<.001
Age, linear	–0.198	0.016	–12.54	<.001
Age, polynomial	–0.100	0.013	–7.83	<.001
Birds as a hobby	0.090	0.036	2.52	.012
Rank of the picture	–0.183	0.015	–12.36	<.001

### 3.2 Detection time of the tawny owl color morphs

All fixed variables were statistically significant (Table 2). Owls were found faster in snowless landscapes than in snowy landscapes. The brown morph was found faster than the gray morph. Brown morph in snowy landscapes was found faster than gray morph in snowy landscapes (Figure 1b). Detection time increased with increasing age of the observer. It took longer to find the owl in pictures that came later in the series. Birdwatchers found the owls faster than non‐birdwatchers.

**Table 2:** LME statistics of a model explaining detection time of owls

	Estimate	*SE*	*t*	*p*
Intercept	1.764	0.046	38.695	<.001
Snow (compared to no snow)	0.090	0.012	7.228	<.001
Color (brown compared to gray)	–0.238	0.012	–20.042	<.001
Color by snow (brown in snow compared to gray in snow)	–0.236	0.017	–13.948	<.001
Age, linear	0.109	0.005	20.804	<.001
Age, polynomial	0.044	0.005	9.591	<.001
Birds as a hobby	–0.081	0.012	–6.898	<.001
Rank of the picture	0.028	0.004	6.326	<.001

### 3.3 Can a human observer tell the color morphs apart?

Of the 390 mounted tawny owl color morph determinations done by the observers, 347 were determined congruently and 43 (21 gray and 22 brown) were determined divergently to color morph scoring done by us using the color scoring method. In 14% of the divergent cases (6/43), one of the model owls was determined wrongly and the remaining 86% of the divergent cases were shared between the other eight mounts. The GLMM showed that the probability to score color correctly was not affected by whether the mount was the same as used in the snow experiment or not, nor was it affected by the color score (Table A1). Color morph was more likely to be scored incorrectly if the picture was taken from the side or the chest than from the back of the owl (Table A1). The picture series or the rank of the picture within a series had no effect (Table A1). We therefore concluded that a human observer can tell the color morphs apart and that the two model mounts are a representative sample from the range of color‐scored mounts and that the angle of the picture adds more variability to the appearance than the mount itself.

### 3.4 Human versus blue tit perception of owl mount coloration

**Figure 2:**
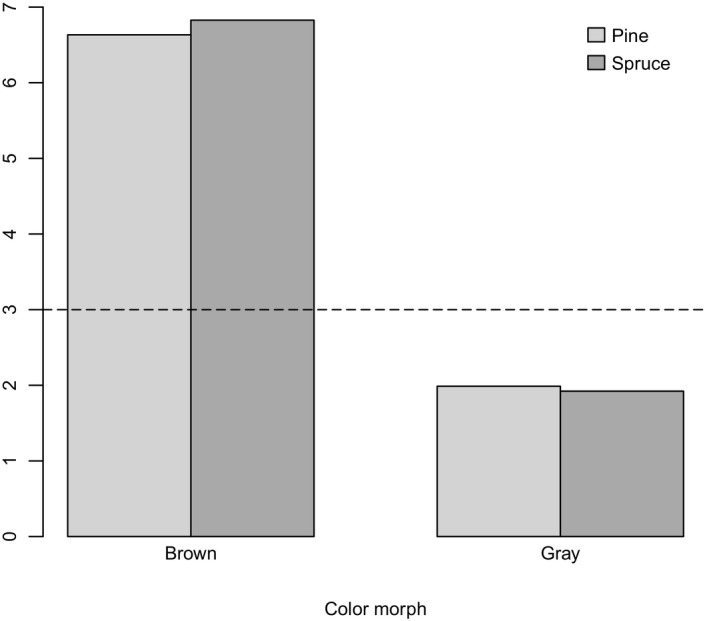
Color contrast JNDs of dorsal sides of brown and gray tawny owl mounts against spruce and pine trunks. The threshold (>3) indicating that trunk and mount are likely discriminable is marked with a dashed line

The avian vision models suggest that both color morphs appear conspicuous in terms of color and luminance for blue tits against green foliage of pine and spruce as well as against snow (JNDs > 3, with the exception of LC JND of dark stripes on the back of gray morph against pine needles). The color contrast values for dorsal side and facial disk of gray tawny owl mount were much lower (<3) against spruce and pine trunks compared to the values for brown tawny owl mount (>6). This indicates that the gray tawny owl can appear more cryptic for blue tits against spruce and pine trunks than the brown tawny owl (Figure 2). In terms of luminance, both morphs appear to be easier to detect from the dorsal side against spruce trunks, compared to pine trunks (Table A2). Difference in color contrast indicates that blue tits are able to tell the two morphs apart even in poor light conditions.

## 4 DISCUSSION

Our results suggest that the gray tawny owl morph could benefit from its coloration via increased crypsis in snowy landscapes. We first showed that, in line with our prediction, the detection probability is lower and detection time is longer for a gray than for a brown tawny owl morph in snowy landscapes. We then demonstrated that this finding was well supported by our vision model analyses: Like for humans, also for birds, the brown morph is more conspicuous against spruce or pine trunks than the gray morph. Our data therefore suggest that a decrease in protectiveness of the coloration could decrease the survival of brown tawny owl morphs in its northern distribution limits and explain why there is strong selection against them in snowy winters whereas this selection is absent in winters with less snow (Karell et al., 2011). These findings are in line with previous studies of tawny owls in lower latitude populations, suggesting that the brown morph is associated with warm and wet environments and the gray one to cool and dry environments (Galeotti & Cesaris, 1996) and that the brown morph, on the other hand, enjoys other fitness benefits over the gray morph in terms of offspring growth (Roulin, Gasparini, Bize, Ritschard, & Richner, 2008) and reproductive success (Emaresi et al., 2014) in favorable food conditions.

In addition to age, also birdwatching experience of the participants had effect on the finding probability and finding time of owls. Birdwatchers are probably more experienced to seek for the owls in potential spots than non‐birdwatchers. As it took longer to find the owl in pictures that came later in the series, we can conclude that there was no learning involved during the series of eight pictures. In contrast, it seems that the enthusiasm and motivation of participants at the beginning of the survey starts to subside to the end. We are confident that the result is not due to unfinished survey attempts as we discarded those attempts. Besides, in addition to finding time, the finding probability also decreased the later the picture came in the series.

Although we acknowledge the inherent issue of using only one individual of each morph in the study design, we argue that the large variation in appearance of the mounts in the different study plots from different angles outweighs the effect of having a desired larger number of specimens (Davies & Gray, 2015). The frequency distribution of tawny owl coloration is bimodal (Brommer et al., 2005; Karell et al., 2011), with peaks in color scores of 6 (gray) and 12–13 (brown). More specifically, in our method validation tests we found that any observer is very likely to determine the color of a mounted owl correctly and that the two model mounts used in the main survey are representative of their morph as they are as likely to be correctly (or wrongly) categorized by an observer as any other mount. On the other hand, it is the angle from which the owl is observed and not the individual mount, which mainly explains how well observers can tease apart the color morphs (Table A1). These results give support for the assumption that the difference in finding probability and detection time of tawny owls in the main survey is because of plumage coloration. We are thus confident that usage of multiple specimens in the main survey would not change the results qualitatively because the morph difference would override possible individual‐specific traits in the plumage color.

Although being broadly acceptable, there is some debate on the accuracy about using human observers when studying the perception of other organisms (Bennett, Cuthill, & Norris, 1994; Bergeron & Fuller, 2018; Endler, 1990; Kemp et al., 2015). We therefore applied an avian vision model based on a blue tit's vision, which suggests that birds' ability to discriminate the tawny owl morphs against their natural backgrounds is along the same lines with the results received from the experiment with humans. The main threats to tawny owls in Southern Finland in winter are resident forest‐dwelling avian predators such as goshawks and eagle owls (*Bubo bubo*), and perhaps foremost the consequences of passerine and corvid mobbing, which indirectly increase a tawny owl's energy demands and exposure to predators. Both humans and potential prey and mobbers of a roosting tawny owl, such as blue tits, are able to detect the brown morph easier than the gray morph against spruce and pine trunks. The avian vision model clearly suggests that it is the chromatic contrast, which makes the brown morph significantly easier to detect compared to the gray in a snowy winter environment. We are thus confident that the approach of using human observers in this case can be justified (Götmark, 1996, 1997; Götmark & Hohifalt, 1995). Future studies should aim at testing the mobbing behavior of passerines in nature, when exposed to the two tawny owl color morphs.

Seasonal variation in physical and visual environments could alter the fitness of gray and brown morphs in several ways. Snow together with coniferous and leafless deciduous trees make the winter landscape “overall gray” and thus may favor the gray tawny owl morph in terms of camouflage. The higher detectability together with potentially poorer plumage insulation capacity (Koskenpato, Ahola, Karstinen, & Karell, 2016) and higher energy requirements (Piault, Gasparini, Bize, Jenni‐Eiermann, & Roulin, 2009) of brown morphs can increase their mortality during harsh and snowy winters. For example, the longer detection time of the gray morph may allow them to prepare themselves for potential encounter with predators and give them time to escape. Also, a lower detection probability can decrease the energy requirements due to movements during harsh time periods and further improve survival probability.

On northern latitudes, especially winters have shortened and are expected to shorten further and faster due to climate change (Diffenbaugh & Field, 2013; Kunkel et al., 2016). Our findings in the color polymorphic tawny owl represent a potentially widespread response to this change. We find that the gray morph is better adapted to bright snowy landscapes whereas this benefit is weak in darker snowless landscapes, as predicted based on previous empirical evidence of selection (Karell et al., 2011). With milder winters and thinner snow cover, the selection periods against the brown tawny owl morph may eventually disappear and the species range margin may reach further north expanding the distribution of, in particular, the gray tawny owl morph. Such cryptic coloration where the color does not match the substrate directly, but only makes coloration cryptic on landscape level, may be a general phenomenon also occurring in forest‐dwelling species with seasonal coat color change. For example, red squirrels (*Sciurus vulgaris*) change coat color from brown in summer to gray in winter, which does not make the individuals directly cryptic with the change in condition (snow layer), but rather may make them more cryptic in the changed light conditions and visual background in the forest landscape. In such cases, it is expected that the choice of microhabitat or roosting sites is color morph‐specific in order to match the background and thereby enhance camouflage. Indeed, recent studies have found morph‐specific fitness effects of variation in light environments in a diurnal raptor (Tate et al., 2016) and in moonlight conditions in a nocturnal owl (San‐José et al., 2019). Future studies should aim at understanding the role of cryptic coloration under environmental change also in species with color variation where cryptic coloration may not be directly linked with a given substrate, but rather linked to variation in overall light conditions and reflectance in the environment.

Our results are in line with the expectations of Gloger's rule (Delhey, 2019) which predicts paler coloration on higher latitudes and in drier and colder environments, and are likely to present a general pattern in nature considering especially species with color polymorphism or seasonal color variation (Mills et al., 2018). For example, in eight seasonal coat color‐changing mammal species the probability of having white coat color is determined by the days of snow cover, which results in a latitudinal cline in seasonal coat color change (Mills et al., 2018). Natural selection may cause rapid evolutionary changes on protective coloration on specific latitudes if climate warming generates a seasonal mismatch between expressed coloration and the environment.

## CONFLICT OF INTEREST

None declared.

## AUTHORS' CONTRIBUTION

KK, PK, and AL designed and conducted the photographing of owl mounts and designed the citizen science survey. KK and AL carried out the statistical analyses. CLK and KK carried out the avian vision modeling. KK wrote the manuscript together with PK, AL, and CLK.

## Figures and Tables

**Figure A1:**
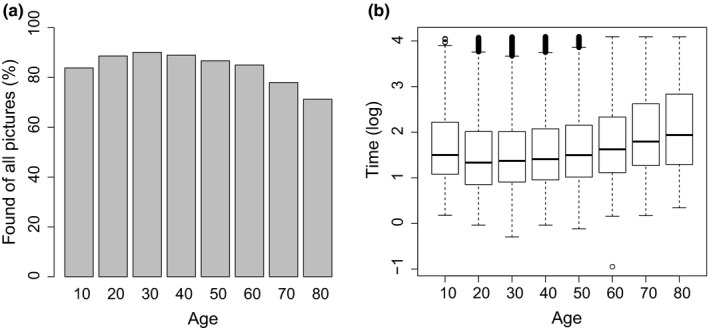
The effect of participant's age on (a) the detection probability and (b) finding time of owls

## Data Availability

All data including study pictures (both main survey and method validation) and scripts are available in Dryad repository https://doi.org/10.5061/dryad.q573n5tf5: Online survey pictures: https://doi.org/10.5061/dryad.q573n5tf5. Validation study pictures: https://doi.org/10.5061/dryad.ttdz08ktc. Data and r code: https://doi.org/10.5061/dryad.2z34tmpgz.

## APPENDIX 1

**Table A1:** GLMM statistics of variables affecting the probability of a human observer to assign color morph correctly

	Estimate	*SE*	*z*	*p*
Intercept	3.684	1.726	2.13	.03
Model mount (compared to not model)	0.146	1.385	0.11	.92
Color score	0.052	0.164	0.32	.75
Angle, chest (compared to back)	–2.661	0.711	–3.74	**<.001**
Angle, side (compared to back)	–2.207	0.732	–3.02	**.003**
Series 2 (compared to Series 1)	1.000	0.571	1.75	.08
Series 3 (compared to Series 1)	0.656	0.597	1.10	.27
Rank of the picture	0.045	0.115	0.39	.69

Note

“Model mount” refers to whether the mount in the picture was used in the online game or not, and “Angle” refers to the angle from which the mount was presented in the picture. Significant *p*‐values (*p* < .05) are highlighted in bold.

**Table A2:** Just noticeable differences (JNDs) of color contrast (CC) and luminance contrast (LC) between different parts of tawny owl color morph plumages against different backgrounds

Plumage part	Background	Brown morph CC JND	Gray morph CC JND	Brown morph LC JND	Gray morph LC JND
Back	Gray morph's back	5.5	NA	0.3	NA
	Pine trunk	**6.6**	**2.0**	**1.0**	**1.3**
	Spruce trunk	**6.8**	**1.9**	**3.5**	**3.8**
	Pine needles	25.9	6.0	14.7	15.0
	Spruce needles	10.2	6.0	15.9	16.2
	Snow	13.2	7.9	25.2	25.1
Facial disk	Gray morph's facial disk	5.3	NA	NA	NA
	Pine trunk	**5.3**	**2.9**	3.2	7.2
	Spruce trunk	**5.2**	**2.7**	5.7	9.7
	Pine needles	8.1	5.4	17.0	20.9
	Spruce needles	8.1	5.7	18.1	22.1
	Snow	11.3	6.2	23.1	19.1
White patches on the face	Brown morph's back	7.5	NA	NA	NA
	Pine trunk	3.6	3.5	14.5	22.1
	Spruce trunk	3.5	3.5	16.9	24.6
	Pine needles	5.9	5.5	28.2	35.8
	Spruce needles	6.3	5.7	29.4	37.0
	Snow	7.1	3.7	11.9	4.3
	Brown morph's white patches	NA	3.7	NA	NA
	Gray morph's back	NA	4.4	NA	20.8
Dark stripes on the back	Gray morph's dark stripes on the back	3.0	NA	2.4	NA
	Gray morph's back	3.3	2.5	10.9	13.2
	Brown morph's back	5.8	7.5	10.6	13.0
	Pine trunk	2.4	1.5	9.6	11.9
	Spruce trunk	2.7	0.8	7.1	9.5
	Pine needles	7.3	5.0	4.1	1.7
	Spruce needles	6.8	4.8	5.3	3.0
	Snow	8.2	5.8	35.9	38.3

Note

JND values <1–3 indicate that the two colors are likely indistinguishable under optimal light conditions, and values >3 indicate that two objects are likely discriminable. Differences in JNDs between morphs and backgrounds are highlighted in bold.
